# Ameloblastic Fibrosarcoma Transformation From Recurrent Ameloblastic Fibroma: A Case Report From Morocco

**DOI:** 10.7759/cureus.93274

**Published:** 2025-09-26

**Authors:** Sanae Soudani, Fatima Safini, Kenza Oqbani, Sanae Abbaoui, Bouchra Amaoui

**Affiliations:** 1 Faculty of Medicine and Pharmacy, Ibn Zohr University, Agadir, MAR; 2 Department of Radiation Therapy, Souss Massa University Hospital, Agadir, MAR; 3 Biomed Laboratory, Faculty of Medicine and Pharmacy, Ibn Zohr University, Agadir, MAR; 4 Department of Pathology, Souss Massa University Hospital, Agadir, MAR

**Keywords:** ameloblastic fibroma, ameloblastic fibrosarcoma, metastasis, rare cancers, recurrence

## Abstract

Ameloblastic fibrosarcoma is an extremely rare odontogenic sarcoma, which consists of a benign epithelial and a malignant mesenchymal component. This rare tumor occurs de novo or from a pre-existing ameloblastic fibroma. We report a case of a 31-year-old male patient who developed a lung metastasis fibrosarcoma secondary to ameloblastic fibrosarcoma arising on a pre-existing mandibular ameloblastic fibroma. A thorough understanding of the radiological and histological aspects of mandibular tumors is essential to establish an accurate diagnosis and rule out other common benign and malignant odontogenic tumors that might display microscopic similarities. An appropriate management, including total surgical excision with safe margins, represents the preferred treatment of ameloblastic fibroma. Close monitoring is indicated to reduce the recurrence rate.

## Introduction

Ameloblastic fibrosarcoma (AFS) is an extremely rare and aggressive odontogenic sarcoma, characterized by a combination of a benign epithelial component, resembling an ameloblastic fibroma (AF), and a malignant mesenchymal component, which is characteristic of a sarcoma.

While AFS constitutes a minority of odontogenic malignancies, it is of particular concern due to its potential for recurrence and metastasis. AFS accounts for approximately 0.3% of all odontogenic tumors and less than 5% of all maxillofacial sarcomas [1,2]. Approximately one-third of AFS cases arise following malignant transformation of a recurrent AF, with the remaining 60% occurring de novo, independent of a preceding AF lesion [3].

Ameloblastic fibroma itself is a benign, mixed odontogenic tumor predominantly seen in the mandibular region, often diagnosed in young adults or adolescents [4]. While AF typically demonstrates a favorable prognosis after surgical resection, recurrence can occur, especially if the lesion is incompletely excised. Malignant transformation of recurrent AF to AFS is a rare but well-documented phenomenon, with various case reports suggesting that inadequate resection or delayed diagnosis may increase the risk of such transformation [5]. This malignant progression is concerning as it can lead to rapid local growth, distant metastasis, and poor patient outcomes.

Diagnostically, AFS poses a challenge due to histopathological similarities with other odontogenic and soft tissue tumors, especially in early stages [6].

Given the rarity of both AF and AFS, the significance of reporting these cases lies in enhancing the understanding of their clinical behavior, improving diagnostic accuracy, and refining treatment strategies. This case report highlights a unique instance of AFS arising in a previously incompletely resected mandibular AF, presenting an opportunity to discuss the challenges in the management of recurrent odontogenic tumors and the importance of thorough surgical intervention.

## Case presentation

**Figure 1:**
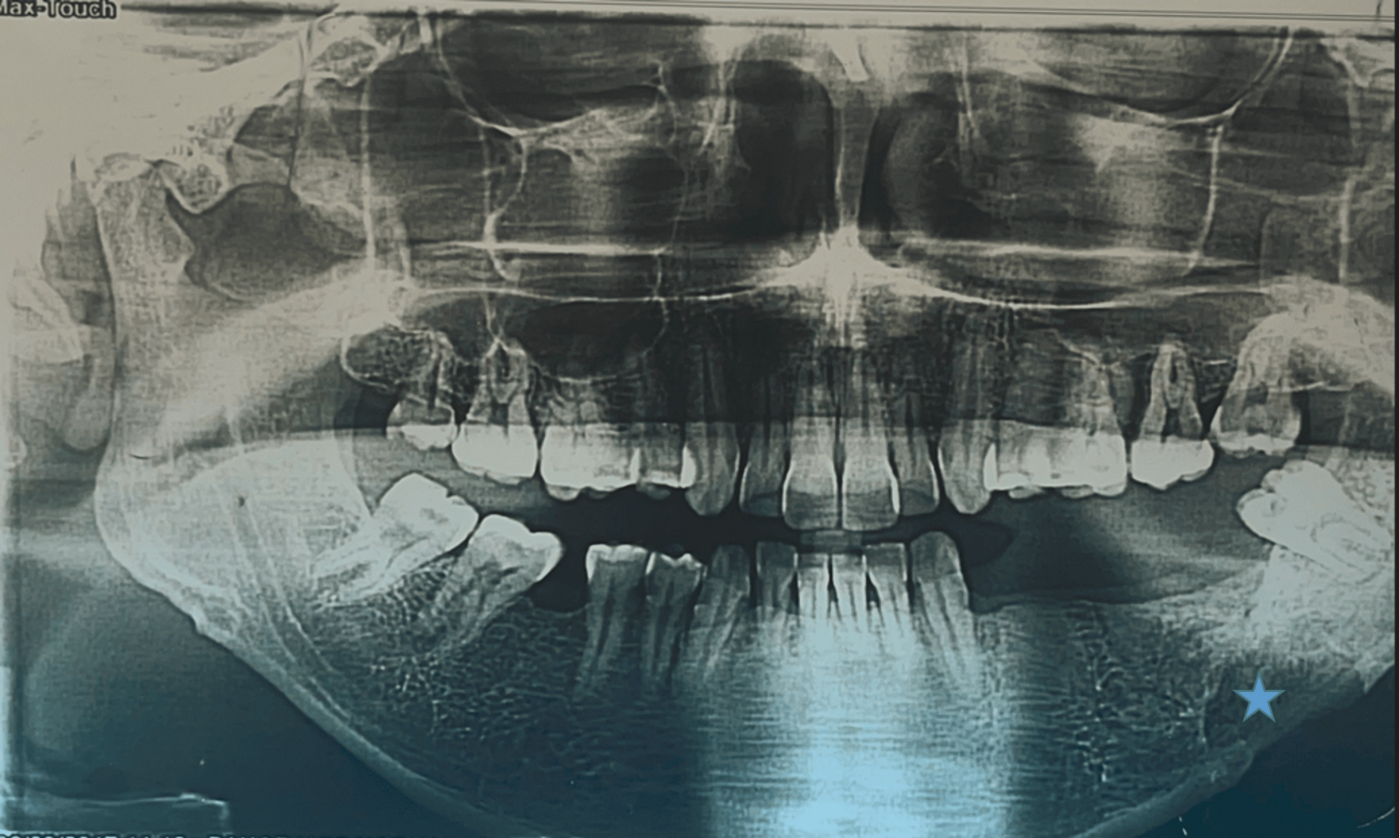
Panoramic X-ray: an irregular lacunar lesion (blue star) at the junction of the horizontal branch and the left mandibular angle, blowing out the cortical bone opposite.

A 31-year-old previously healthy male presented in 2017 with gingival swelling following a dental extraction. Clinical examination found a mass in the left mandibular angle region. A panoramic radiograph showed an irregular lacunar lesion at the junction of the horizontal ramus and the left mandibular angle (Figure 1).

Based on clinical and radiographic findings, the initial differential diagnoses included odontogenic keratocyst, central giant cell granuloma, and ossifying fibroma. However, histopathological examination of the excised lesion confirmed the diagnosis of an aggressive ameloblastic fibroma, with a Ki-67 proliferation index of approximately 15%. The status of the surgical margins was not documented.

**Figure 2:**
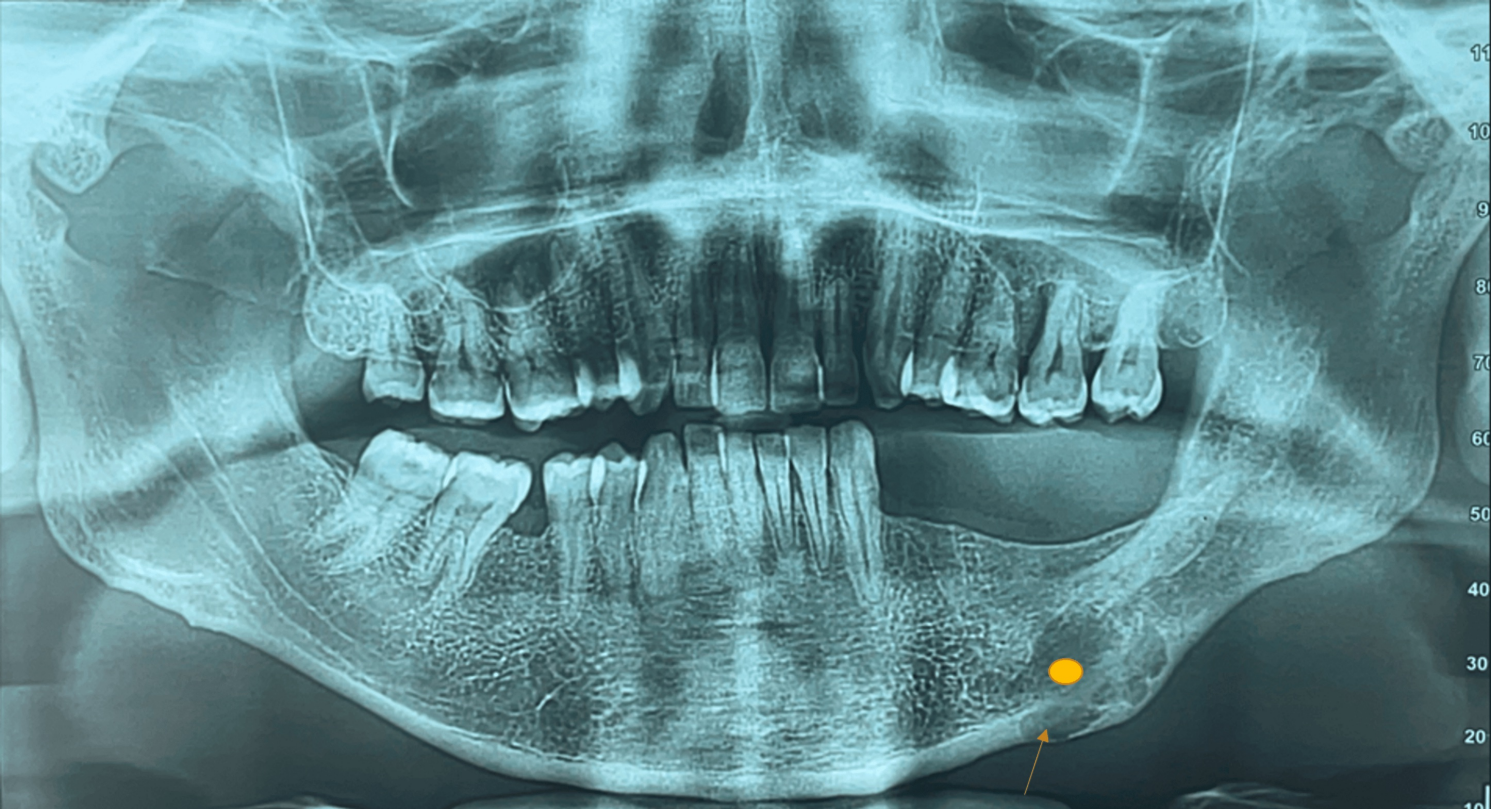
Panoramic X-ray: a lytic lesion at the junction of the horizontal branch and the left mandibular angle with lobulated contours (orange circle), with local cortical thinning and interruption (red arrow).

**Figure 3:**
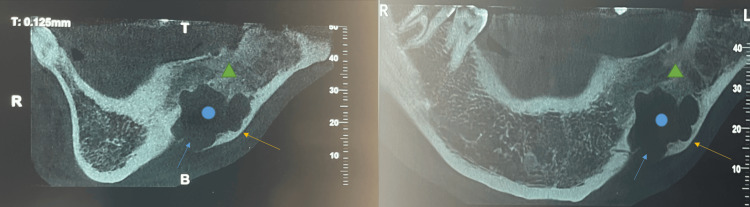
Sagittal (A) and coronal (B) complement scans, showing an irregular lytic patch at the junction of the horizontal branch and the left mandibular angle (blue circle), with cortical thinning (orange arrow) and cortical interruption (blue arrow) associated with adjacent bone infiltration reaching the mandibular alveolar process (green triangle).

Six months later, the patient experienced local recurrence at the same site. A panoramic X-ray was performed, revealing a unilocular osteolytic expansive lesion of the left mandibular ramus with cortical bone rupture (Figure 2). A subsequent CT scan of the mandible demonstrated a multilocular lytic lesion of the left horizontal mandibular branch, measuring 4 × 2.7 × 1.5 cm, extending into the adjacent alveolar bone. The patient underwent wide surgical excision of the lesion (Figure 3A, 3B), and pathological analysis confirmed a recurrent ameloblastic fibroma. As with the initial surgery, the surgical margin status was not specified in the pathology report.

The patient remained asymptomatic for three years. In 2020, he presented again with a rapidly enlarging, necrotic gingival mass involving the left mandible. Imaging and clinical evaluation led to a decision to perform a wide hemimandibulectomy. Histological analysis of the surgical specimen revealed a high-grade fibrosarcoma, Fédération Nationale des Centres de Lutte Contre le Cancer (FNCLCC) grade III, with a positive posterior margin.

One month after surgery, the patient exhibited local tumor progression with a mass measuring 8 × 3.9 cm, infiltrating both the pterygoid and masseter muscles. Given the inoperability of the recurrent lesion, the case was discussed in a multidisciplinary tumor board, and radiotherapy was indicated. The patient received external beam radiation therapy to the local site, with a total dose of 70 Gy delivered in 35 fractions of two Gy each.

**Figure 4:**
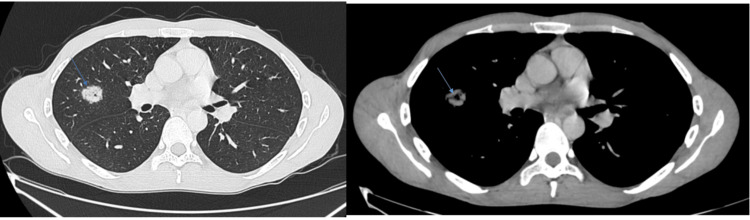
Parenchymal (A) and mediastinal (B) axial scans showing a nodule in the right upper lobe, in the early stages of excavation, with irregular contours and tissue contrast.

**Figure 5:**
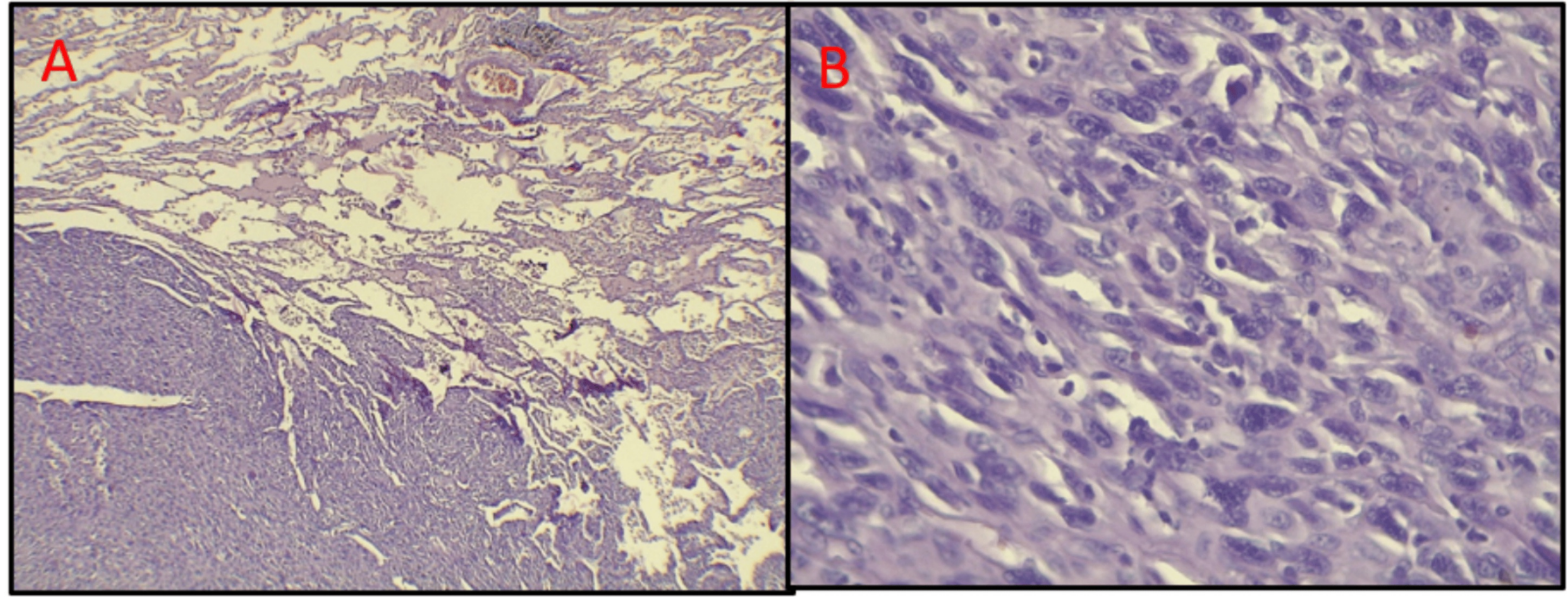
Microscopic images of the lung metastasis: the microscopic images revealed malignant mesenchymal proliferation invading the pulmonary parenchyma (H&E x.4) (A). Fusocellular proliferation demonstrated pleomorphic spindle tumor cells favoring fibrosarcoma (H&E x.40) (B).

He remained disease-free for 18 months. However, follow-up thoracic CT performed as part of routine surveillance revealed a solitary nodule in the right upper lobe (Figure 4A, 4B). CT-guided biopsy confirmed the diagnosis of pulmonary metastasis of fibrosarcoma, with no evidence of the epithelial ameloblastic component (Figure 5A, 5B). A right upper lobectomy was performed, and the patient has been under close clinical and radiological follow-up since. At the 18-month postoperative mark, the patient remains in complete remission.

## Discussion

Ameloblastic fibrosarcoma was first described by Heath in 1887 as a spindle cell sarcoma containing epithelial cells resembling the enamel organ [7]. It is a rare odontogenic malignancy that must be distinguished from other odontogenic neoplasms for proper management.

Historically, terms such as ameloblastic dentinosarcoma and ameloblastic odontosarcoma have been used for odontogenic neoplasms containing dentin or enamel, respectively. However, in the 5th edition of the World Health Organization (WHO) classification of odontogenic tumors, ameloblastic odontosarcoma and dentinosarcoma are listed separately from the AFS [8].

AFS can occur in patients ranging from three to 89 years old, with a male predominance. Among 103 documented cases, 71 arose de novo and 25 developed from recurrent AF, as seen in our case. Secondary AFS often occurs in patients with a prior histologically confirmed AF at the same site. The mean age for AFS diagnosis is generally higher than that for AF [8-10].

Michelle et al. proposed that malignant transformation involves a stepwise accumulation of genetic mutations, with p53 overexpression playing a significant role [11].

Clinically, AFS typically presents as a painful mandibular mass, often associated with paresthesia. Our case presented initially as painless gingival swelling. Radiographically, AFS usually appears as an ill-defined, radiolucent, and expansile lesion with cortical bone involvement

Grossly, the tumor may be solid or cystic, often destroying adjacent bone. Microscopically, it consists of benign odontogenic epithelium arranged in nests or cords, and a malignant mesenchymal component composed of spindle cells with pleomorphism, high mitotic activity, and sometimes necrosis [12]. The FNCLCC grading system is used to evaluate sarcomatous components based on mitotic index, tumor necrosis, and differentiation [5].

Fibrosarcoma and other spindle-cell sarcomas are important considerations in the differential diagnosis of AFS. A key distinguishing feature lies in the absence of ameloblastic epithelium in the former, whereas it is typically present in AFS, particularly those arising from ameloblastic fibroma. However, multiple reports have noted that the epithelial component may progressively diminish or even disappear entirely as malignant transformation advances [2].

The histopathological evolution in our case - from AF to AFS, and eventually to pulmonary fibrosarcoma - mirrors the progression described by Leider et al., who noted loss of the epithelial component in later recurrences and considered it a degenerative phenomenon [13]. This was similarly observed in our patient’s lung metastasis, where the epithelial ameloblastic component was absent (Figure 5A, 5B).

There is debate in the literature regarding the metastatic potential of AFS. Prein et al. described it as a “semi-malignant” lesion due to its generally local behavior and proposed the term proliferative ameloblastic fibroma [14]. However, other reports, including that of Chomete et al., describe distant metastasis to the liver and mediastinal lymph nodes [15]. Our case further supports the malignant potential of AFS by demonstrating confirmed lung metastasis.

## Conclusions

Although rare, AFS should be considered in the differential diagnosis of recurrent ameloblastic fibroma, especially when malignant transformation is suspected. The treatment of choice is wide surgical excision with negative margins, supported by long-term follow-up to monitor for recurrence and metastasis. In selected cases, particularly those that are unresectable or advanced, radiation therapy and, less commonly, chemotherapy may be considered. Optimal management requires a multidisciplinary approach to tailor treatment and improve patient outcomes.
